# Managing Rhizoctonia Damping-Off of Rocket (*Eruca sativa*) Seedlings by Drench Application of Bioactive Potato Leaf Phytochemical Extracts

**DOI:** 10.3390/biology9090270

**Published:** 2020-09-04

**Authors:** Catello Pane, Michele Caputo, Gianluca Francese, Gelsomina Manganiello, Roberto Lo Scalzo, Giuseppe Mennella, Massimo Zaccardelli

**Affiliations:** 1 Consiglio per la Ricerca in Agricoltura e l’Analisi dell’Economia Agraria, Centro di Ricerca Orticoltura e Florovivaismo, via dei Cavalleggeri 25, I-84098 Pontecagnano Faiano, Italy; caputomichele1986@libero.it (M.C.); gianluca.francese@crea.gov.it (G.F.); manganiellogelsomina@gmail.com (G.M.); giuseppe.mennella@crea.gov.it (G.M.); massimo.zaccardelli@crea.gov.it (M.Z.); 2 Consiglio per la Ricerca in Agricoltura e l’Analisi dell’Economia Agraria, Centro di Ricerca Ingegneria e Trasformazioni Agroalimentari, via Venezian 26, I-20133 Milano, Italy; roberto.loscalzo@crea.gov.it

**Keywords:** antifungal activity, antioxidant activity, plant pathology, plant secondary metabolites, *Rhizoctonia solani*, *Solanum tuberosum*, sustainable agriculture

## Abstract

Plants produce a huge array of secondary metabolites that play a key role in defense mechanisms against detrimental microorganisms and herbivores, and represent a suitable alternative to synthetic fungicides in sustainable agriculture. In this work, twelve crude hydroethanolic extracts derived from leaves of different potato cultivars were chemically characterized by LC/MS and their antioxidant properties were investigated in vitro. Furthermore, the biological activity against the fungal pathogen *Rhizoctonia solani* was evaluated both in vitro and in vivo. Extracts showed the ability to inhibit *R. solani* growth in vitro and significantly reduced damping-off incidence in in vivo experiments. Furthermore, *R. solani* mycelia exposed to the extracts showed an altered morphology (low translucency, irregular silhouette, and cytoplasmatic content coagulation) compared to the untreated control in light microscopy examination. Principal component analysis conducted on identified chemical compounds highlighted significant metabolic variations across the different extracts. In particular, those that inhibited most of the growth of the pathogen were found to be enriched in α-chaconine or α-solanine content, indicating that their biological activity is affected by the abundance of these metabolites. These results clearly indicated that plant-derived compounds represent a suitable alternative to chemicals and could lead to the development of new formulates for sustainable control of plant diseases.

## 1. Introduction

Damping-off caused by several soil-borne pathogens, including the fungus *Rhizoctonia solani* Kühn [teleomorph, *Thanatephorus cucumeris* (Frank) Donk], is a major constraint in many sown vegetable crops. The pathology is common of sick and intensively exploited soils such as those cultivated for baby-leaf rocket production, causing germinated seed death or seedlings collapse, bringing about direct economic consequences [1]. Several chemical, physical, and ecological soil disinfection approaches are proposed to keep down the telluric population of *R. solani* [2]. However, the full control of the pathogen is complicated by its inherent aptitude to the saprophytic lifestyle, the production of conservative structures (i.e., pseudosclerotia), favorable environmental factors (i.e., high crop density, soil moisture, temperate conditions), and the wide host range that promote survival into the soil [3]. Furthermore, the drawbacks related to the use of chemical fungicides, such as undesirable non-target effects, fungal resistance, and other sustainability reasons are inciting the progressive reduction of many chemical soil disinfectants and fungicides from disease management protocols [4]. Therefore, the introduction of new eco-friendly improved non-synthetic plant protection measures is encouraged. According this perspective, many antifungal phytochemicals isolated from higher plants have developed in the last years [5]. They are harmless and non-toxic compounds such as alkaloids, polyphenols, and saponins with remarked antimicrobial properties that make some plant extracts promising for the treatment of multiple plant diseases including the seedling damping-off [6].

Seed-soaking treatments with phytochemical blend extracted from *Thespesia populnea* and *Chrysanthemum frutescens* were found effective, for example, against the sugar beet damping-off caused by *Sclerotium rolfsii* [7]. El-Mougy and Abdel-Kader [8] showed a reduction of faba bean damping-off caused by *Fusarium solani* and *R. solani* by seed dressing with powder or extract of carnation, garlic, cinnamon, or thyme. Lupine seeds treated with extracts of *Nerium oleander* and *Eugenia jambolana* leaves and *Citrullus colocynthis* fruits significantly reduced damping-off disease caused by *Fusarium oxysporum* f. sp. *lupini* [9].

Medicinal plants have often been selected for the phytochemicals extraction [10]; however, the residues of cultivated plants have been also investigated for the production of raw antifungal extracts in a view of circular economy and agricultural waste valorization. Examples of non-edible plant parts used as source of phytochemicals for anti-pathogenic applications were reported for pepper and eggplant leaves [11,12,13], chestnut burs [14], and/or grape waste [15]. Plant secondary metabolites have a key role in biotic and abiotic stress responses, in allelopathy mechanisms and in plant-plant and plant-microbes interaction [16]. Although the presence of these compounds have genetic basis, their production and accumulation are strongly modulated by environmental factors (i.e., light, temperature, soil fertility, salinity) [17]. The main aim of this work was to study the effect of twelve hydroethanolic extracts derived from leaves of different potato cultivars against the fungal pathogen *R. solani*. The biological activity of the phytochemical mixtures was investigated both in vitro and in vivo, evaluating the inhibition of pathogen growth and the incidence of damping-off, respectively. Furthermore, in order to link the biological activity to the phytochemical composition, extracts were chemically characterized by LC/MS, and for their in vitro antioxidant properties by Folin–Ciocalteu phenol index and 2,2-diphenyl-1-picrylhydrazyl (DPPH) scavenging.

## 2. Materials and Methods

### 2.1. Plant Materials and Extracts Preparation

Potato cultivars Agria (Ag), AR-03-3410 (AR), Hansa (Ha), Jazzy (Ja), Luminella (Lu), Melody (Md), Melrose (Mr), Perline (Pe), Piatlina (Pi), Piccolo Star (PS), Postiglione (Po), and Ricciona di Napoli (Ri) were cropped in open field at density of 5 tubers m^−2^. Fresh leaves were collected when plants reached the stage of full flowering identified by 2-digit code 69/7N on the BBCH (Biologische Bundesantalt, Bundessortenamt and CHemische Industrie, Germany) scale [18] and dried at 70 °C until constant weight. Plant materials were ground to fine powder and extracted by soaking 10 g in 100 mL ethanol (50% vol.) for 24 h at room temperature. The mixtures were filtered (Whatman Grade 1 filter paper, GE Healthcare Life Sciences, Uppsala, Sweden), and the solvent evaporated under vacuum (rotary evaporator at 30 °C, Heldolph, Schwabach, Germany). Dry extracts were dissolved in distilled water at concentration of 200 mg mL^−1^, and after filter-sterilization (filter 0.22 µm pore size, Merck Millipore Ltd., Darmstadt, Germany), were stored at −20 °C until use.

### 2.2. Fungal Pathogen

The virulent isolate of the fungal pathogen *R. solani* AG-4 used in this study, was obtained from the fungal collection of the CREA Centro di Ricerca Orticoltura e Florovivaismo (Pontecagnano Faiano, Italy), and maintained on potato dextrose agar (PDA, Difco Laboratories, Detroit, MI, USA) slants at 20 °C. The stock cultures were kept at 4 °C. 

### 2.3. In Vitro Determination of the Antifungal Activity

The antifungal effects of the foliar potato extracts on the in vitro radial growth of *R. solani*, was determined by the poisoned food method. Crude extracts were amended into PDA (0.1×) to obtain the final concentration of 20 mg mL^−1^, and then 2 mL were poured into Petri dishes of 3 cm diameter. A fungal disc of 0.5 mm diameter obtained from actively growing culture were placed upside down on the center of each Petri plate; while, not amended ones were used as reference control; then, plates inoculated in triplicate, were incubated in the dark at 25 °C. At 48 h, when the fungus reached the edge of the control plates, the radius (R) of each colony was measured. The experiment was repeated.

The percentage of fungal growth inhibition was calculated with the following formula:

Inhibition (%)= 100 × [R(control) − R(treatment)]/R(control) (1)

Furthermore, whenever possible, the effective concentration causing 50% of growth inhibition (EC50) was determined from dose-relative inhibition curves obtained from PDA dishes amended with concentrations in a range between 5 and 20 mg mL^−1^ prepared, according with the same procedure reported above. 

To investigate the effects of the antifungal extracts on the fungal morphology, twenty plugs (0.5 mm diameter) of fungal culture were inoculated into 100 mL potato dextrose broth (PDB—Difco Laboratories, Detroit, MI, USA), and incubated at 25 °C in agitation for 24 h. Then, about an mg of growing mycelium was recovered, and inoculated into distilled sterile water supplemented with 20 mg mL^−1^ of extract. After further 24 h, mycelium was took, stained with trypan blue and examined under light microscope (Nikon Eclipse 80i, Nikon, Melville, NY, USA) at 40× magnification. Hyphae from not enriched-distilled sterile water were used as not-treated reference control. 

### 2.4. In Vivo Estimation of Damping-Off Control

The in vivo bioassay was carried out in nursery trays previously filled with sterile peat inoculated (0.5% w/w) with *R. solani* infected millet prepared according to Pane et al. [19]; whereas, sterile peat alone was used for the health control. Seeds of rocket (*Eruca sativa* Mill.) were sterilized by soaking them in 1% sodium hypochlorite for 2 min, washed thoroughly in sterile distilled water, dried on blotting paper, and sown in nursery trays considering five seeds per hole with five replicates per treatment. Each inoculated halo was pre-treated by pipetting 1 mL of extract at a concentration of 20 mg mL^−1^ while, sterile distilled water was used in the non-treated infected control. Then, the tray was incubated in a climatic chamber at 25 ± 2 °C. A week after inoculation, damping-off incidence was assessed by counting the survived seedlings. The experiment was carried out two times. 

### 2.5. Total Phenol Content and Free Radical Scavenging Capacity

The foliar potato hydroethanolic extracts were used for the evaluation of in vitro antioxidant capacity, measured by the scavenging of a stabilized free radical, the 2,2-diphenyl-1-picrylhydrazyl (DPPH). This assay was performed following the rationale of Brand-Williams et al. [20] and Foti [21], by measuring the DPPH extinction for 60 s of reaction at 25 °C. The DPPH assayed solution was freshly made for each analytical session, with the DPPH powder dissolved in absolute ethanol and sonicated for 10 min before use. The reaction solution was composed of 300 μL of MeOH, 50 μL of a 0.5 mM DPPH solution, and 100 μL of potato extract 10-fold diluted with MeOH.

The reaction of DPPH was monitored by Electron Paramagnetic Resonance (EPR) using a MiniScope MS200 Magnettech (Berlin, Germany) and the EPR signal abundance of the assayed potato extracts were compared with those of a system reaction with the extraction solvents in the absence of plant extracts. The experimental conditions of the spectrometer were: field set, 3349.50 G; scan range, 68 G; scan time 60 s, modulation amplitude, 3000 mG; microwave attenuation, 4 dB; receiver gain, 2 × 100.

The percent scavenging was calculated by the following equation:

%SCAV = 100 − [(ABs/ABx) × 100](2)

where ABs is the abundance of the main EPR signal of DPPH spectrum (3349.77 G) of the sample extract and ABx is the related signal of the system reaction in absence of potato extract. The system was calibrated with ascorbic acid (AsA) solution at known concentrations, treated in the same way as the samples, so obtaining a linear dose-effect response (f(x) = 220 X + 27.3, R2 = 0.984). The final data units for DPPH scavenging have been given in equivalents of AsA per mL of potato extract by the interpolation of percent scavenging of the potato extracts vs. the calibration curve. Potato samples were assayed in duplicate.

The colorimetric analysis of total phenolic content of the extract was determined following the Folin–Ciocalteu assay [22]. An aliquot of 100 μL of extract was diluted with 5 mL distilled H_2_O and 0.5 mL of 2M Folin–Ciocalteu reagent (Sigma-Aldrich, St. Louis, MO, USA) and left at room temperature for 5 min. The solution was then treated with 1 mL of 15% Na_2_CO_3_ and incubated in the dark for 1 h. The absorbance was measured at 730 nm with a JASCO spectrometer. The indexes of total phenolic content were estimated by comparison with the standard curve obtained with known concentrations of gallic acid (y = 2.202 x; R2 = 0.99) and expressed as milligrams of gallic acid equivalents per mL of potato extract (mg GAE mL^−1^). Each analysis was performed in duplicate. 

### 2.6. Phytochemical Analysis of Extracts

All the extracts were analyzed in triplicate using reversed phase liquid chromatography coupled to a photodiode array detector and to an ion trap mass spectrometry (LC–PDA–MS) system as described by Docimo et al. [23] with slight modifications. The system consisted of an ultra-performance liquid chromatography (UPLC) Dionex UltiMate 3000 model coupled to an LTQ XL (liner triple quadrupole XL) mass spectrometer (Thermo Fisher Scientific, Sunnyvale, CA, USA). A 100 μL aliquot of sample was diluted with water LC/MS grade (1:10), and then, 4 μL were injected onto a Luna C18 (100 × 2.0 mm, 2.5 μm particle size) column equipped with a SecurityGuard guard column (3.0 × 4.0 mm) (Phenomenex, Torrance, CA, USA). The separations were carried out using a binary gradient of ultrapure water (A) and acetonitrile (B), both acidified with 0.1% (v/v) formic acid, with a flow rate of 0.22 mL min^−1^. The initial solvent composition was 95% (v/v) of A and 5% (v/v) of B; varied linearly to 25% A and 75% B in 25 min and maintained for 1 min; returned to 95% of A in 1 min. The column was equilibrated to 95% A and 5% B for 11 min before the successive injection. The analysis lasted for 38 min and the column temperature was set at 40 °C. Mass spectra were obtained in positive ion mode over the range m/z 80–1400. The capillary voltages were set at 9.95 V and the source temperature was 34 °C. The putative identification of some metabolites was confirmed by m/z data from authentic, distinct standards such as chlorogenic acid (CA), α-solanine (SN), tryptophan (TRP) and isoquercitrin (IS) (Sigma-Aldrich, St. Louis, MO, USA). Solasodoside A (SDA), N-feruloylputrescine (FP), and α-chaconine (CN) were tentatively identified based on MS/MS fragments identity and retention time (r.t.) by matching the resulting spectra with those reported in *S. tuberosum* [24,25,26] and *Solanum melongena* L. [12,27,28] secondary metabolite databases. Xcalibur software (Thermo Fisher Scientific, Sunnyvale, CA, USA) was used to control all instruments and for data acquisition and data analysis. 

### 2.7. Data Analysis

Data about the effects of the extracts on the pathogen development and on the control of the disease were analyzed using the software GraphPad Prism. Since no significant experiment effects were recorded, data from repeated experiments were combined for the analysis. Multiple comparisons were performed by one-way analysis of variance (ANOVA) followed by Bonferroni correction (*p* ≤ 0.05). In order to determine the extent of differentiation among the extracts in relation with their antifungal activity, all the analyzed characteristics (abundance of identified compounds and antifungal activity) were subjected to principal component analysis (PCA), multiple variable analysis by Pearson correlation matrix and hierarchical clustering by ClustVis software (https://biit.cs.ut.ee/clustvis/). Data were normalized using the unit variance scaling; the Singular Value Decomposition (SVD) with imputation was the PCA method applied. Both rows and columns in the heatmap were clustered using correlation distance and average linkage. Pearson correlation between extract bioactivities and the percentage of CN on the total amount of the two glycoalkaloids. Pearson’s coefficients were calculated for biochemical parameters from a regression of cultivar mean values using the statistical software JMP v7.0 [29]. 

## 3. Results

### 3.1. In Vitro Antifungal Activity of Extracts

**Figure 1:**
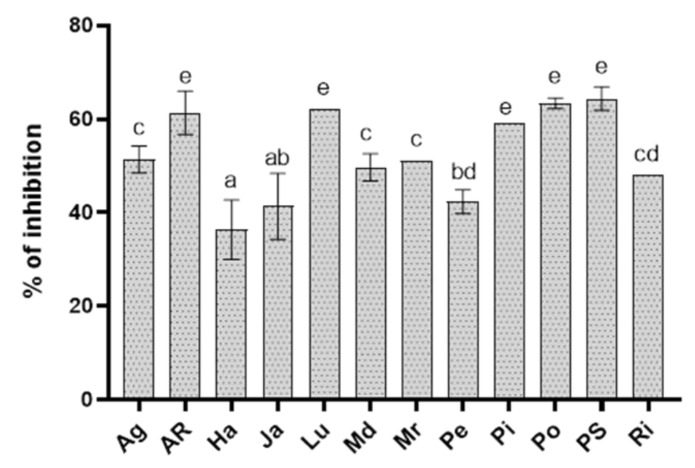
Percentage of inhibition of *Rhizoctonia solani* growth in vitro by potato cv Agria (Ag), AR-03-3410 (AR), Hansa (Ha), Jazzy (Ja), Luminella (Lu), Melody (Md), Melrose (Mr), Perline (Pe), Piatlina (Pi), Piccolo Star (PS), Postiglione (Po), and Ricciona di Napoli (Ri) foliar extracts applied at the dose of 20 mg mL^−1^. Bars are the mean values ± standard error. Data were analyzed with one-way ANOVA; different lowercase letters indicate significant differences among means according with Bonferroni correction for multiple comparisons (*p* ≤ 0.05).

The inhibitory effect of hydroethanolic extracts assayed at the highest concentration (20 mg mL^−1^) on the radial growth of *R. solani* is showed in Figure 1. Among these, AR, Lu, Pi, Po, and PS extracts showed the highest inhibitory activity against the phytopathogen *R. solani* with EC50 values ranging between 3 and 14 mg mL^−1^ (Table 1). All the other extracts had moderate activity against the fungus.

**Figure 2:**
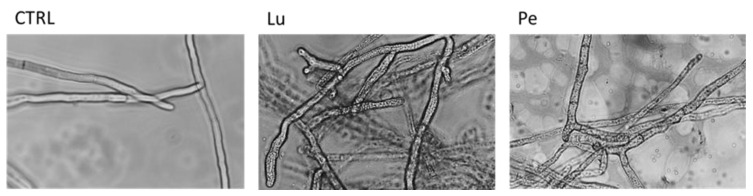
Light micrograph of *Rhizoctonia solani* hyphae exposed to 20 mg mL^−1^ of Luminella (Lu) and Perline (Pe) potato foliar extracts in comparison to the untreated control (CTRL).

The light microscopy examination of *R. solani* mycelia exposed to the Lu and Pe extracts showed an altered morphology compared to the smooth and viable mycelia of the control (Figure 2). Hyphal diameter was measured and resulted significantly larger under Lu (15.55 ± 2.59 µm) and Pe (14.49 ± 0.66 µm) exposure than in the control (11.77 ± 1.08 µm). Treated hyphae have lost their translucency, displaying barrel walls, irregular silhouette, and coagulation of the cytoplasmic content. In addition, treated hyphae were shorter and slightly more branched than control.

### 3.2. Control Assays of Rocket Seedling Damping-Off

**Figure 3:**
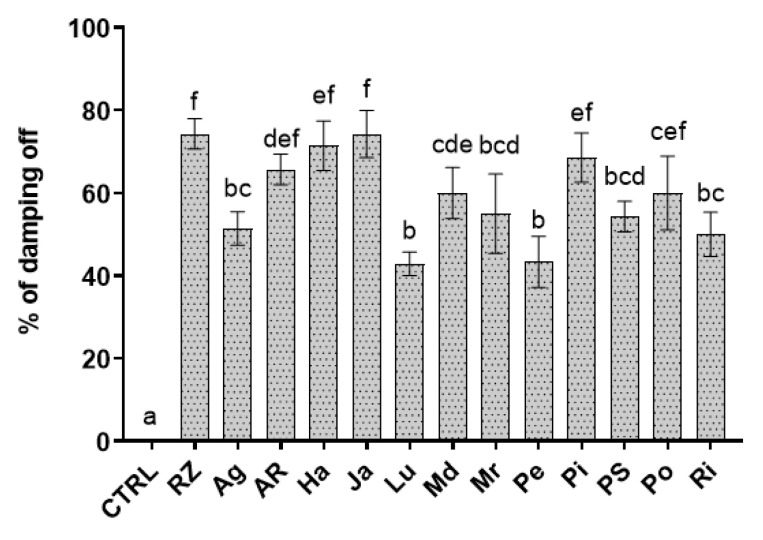
Effect of potato cv Agria (Ag), AR-03-3410 (AR), Hansa (Ha), Jazzy (Ja), Luminella (Lu), Melody (Md), Melrose (Mr), Perline (Pe), Piatlina (Pi), Piccolo Star (PS), Postiglione (Po), and Ricciona di Napoli (Ri) foliar extracts on the percentage of Rhizoctonia damping-off, recorded a week after sowing, in comparison with the non-treated healthy control (CTRL) and the only infected one (RZ). Bars are the mean values ± standard error. Data were analyzed with one-way ANOVA; different lowercase letters indicate significant differences among means according with Bonferroni correction for multiple comparisons (*p* ≤ 0.05).

The nursery-tray assays assessed the efficacy of the extracts against rocket damping-off caused by *R. solani*, and showed a considerable variable effect among the treatments. Lu and Pe extracts were the most effective in reducing damping-off incidence at levels lower than 50% compared to the infected control. These are followed by the cluster including Ag, Md, Mr, PS, and Ri extracts, which exhibited an intermediate efficacy by reducing rocket seedling disease around 51–60% of the infected control (Figure 3). The group with AR, Ha, Ja, Pi, and Po did not show any significant differences with the non-treated infected control.

### 3.3. Metabolite Profiling of Extracts

**Figure 4:**
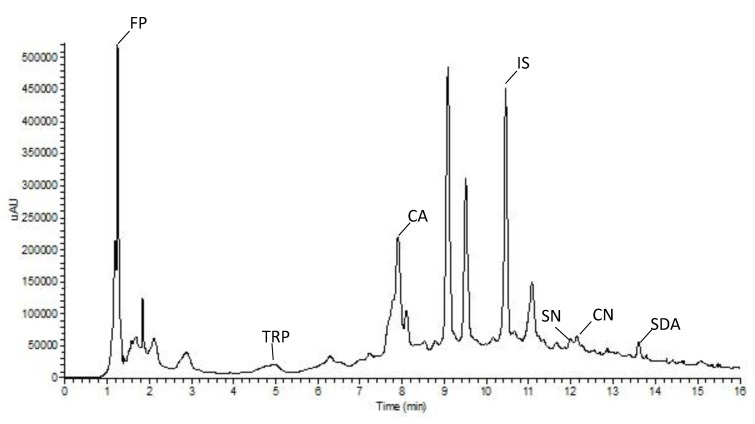
Typical UPLC chromatogram (time range = 0–16 min) of a *Solanum tuberosum* hydroethanolic foliar extract, in which peaks of n-feruloylputrescine (FP), tryptophan (TRP) chlorogenic acid (CA), isoquercitrin (IS), α-chaconine (CN), α-solanine (SN) and solasodoside A (SDA) are indicated.

As expected, the analysis of chromatograms highlighted different metabolic profiles in the twelve extracts analyzed; a typical UPLC profile is reported in Figure 4. The quantitative analysis evidenced the highest amount of CA in the cultivar Ri (8.37 µg mL^−1^) whereas the lowest one was detected in Mr cultivar (5.1 µg mL^−1^); as regards SN content, the cultivar Po and the cultivar Ja showed the highest (749.5 µg mL^−1^) and the lowest (200.3 µg mL^−1^) amount, respectively (Table 2). 

Significant differences were evidenced in CA and SN concentrations among the foliar extracts of the twelve potato varieties studied (Table 2). Differentially present semi-polar metabolites were revealed by LC-MS analysis in these extracts (Table 3) and tentatively identified as: i. FP, a conjugated polyamine (r.t. 0.96, m/z = 265); ii. TRP, an amino acid (r.t. 5.06, m/z = 205; iii. CA, a monomeric phenol (r.t. 7.97, m/z = 355); iv. IS, a flavonoid (r.t. 10.90, m/z = 465); v. SN and CN two glycoalkaloids (r.t. 12.04 and m/z = 869, r.t. 12.20 and m/z = 852, respectively); and vi. SDA, a saponin (r.t. 14.04 and m/z = 1031). The semi-quantitative analysis showed for Po cultivar a higher amount of FP, TRP, SN, and CN compared to the other cultivars. IS peaked in Ha foliar extract whereas PS sample showed the highest amount of SDA (Table 3).

### 3.4. Biochemical Correlations

The statistical relationships between biochemical compound levels in leaf material of the different *S. tuberosum* genotypes studied are reported in a correlation matrix (Table 4). Most biochemical traits positively correlated with each other, with the exception of SDA, which negatively but not significantly correlated to CA, SN, IS, and TRP; CA positively and significantly correlated to SN, IS, and TRP (*p* < 0.01) and to CN and FP (*p* < 0.05). The strongest positive correlations were found both between the glycoalkaloids CN and SN, and between CA and TRP (0.74). Strong positive correlations were also detected between each glycoalkaloid and FP, and TRP, respectively.

### 3.5. Total Phenol Content and Antioxidant Activity of Extracts

**Figure 5:**
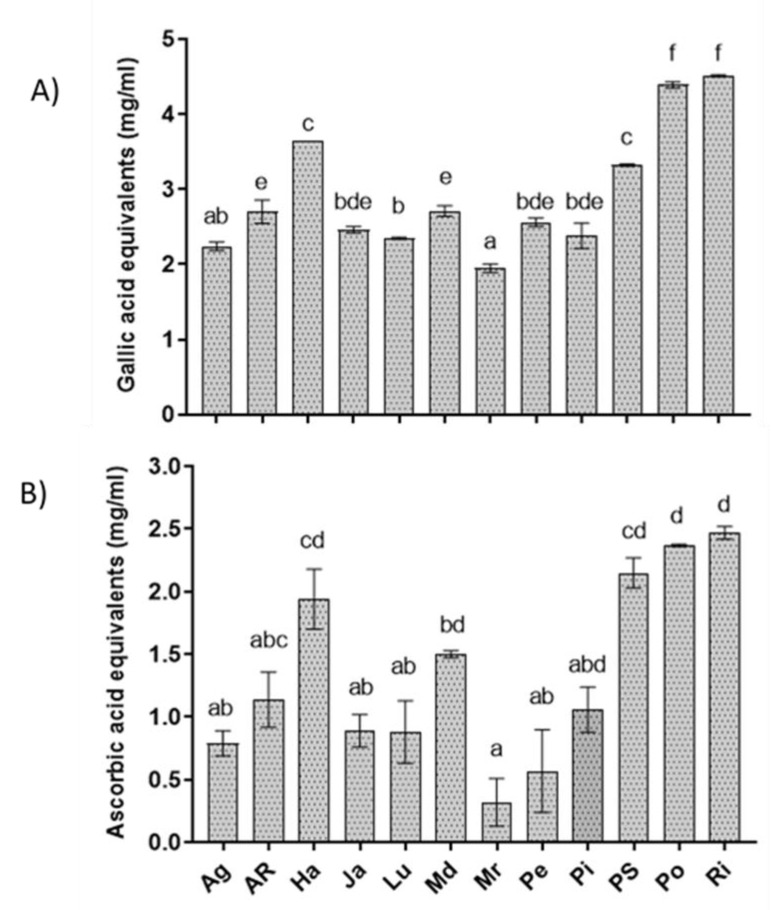
Total phenol content (**A**) and antioxidant activity (**B**) of potato cv Agria (Ag), AR-03-3410 (AR), Hansa (Ha), Jazzy (Ja), Luminella (Lu), Melody (Md), Melrose (Mr), Perline (Pe), Piatlina (Pi), Piccolo Star (PS), Postiglione (Po), and Ricciona di Napoli (Ri) foliar extracts. Bars are the mean values ± standard error. Data were analyzed with one-way ANOVA; different lowercase letters indicate significant differences among means according with Bonferroni correction for multiple comparisons (*p* ≤ 0.05).

The results revealed that Po, Ri, PS, and Ha had the highest content of phenolic compounds among the tested extracts (4.5, 4.39, 3.32 and 3.65 mg GAE mL^−1^, respectively) and also the highest free radical-scavenging activity assessed as AsA abundance (2.37, 2.47, 2.15, 1.94 mg AsA mL^−1^, respectively) (Figure 5). All the other extracts showed lower phenolic and AsA content, with values between 2 and 3 mg mL^−1^ for GAE and between 0.3 and 1.5 mg mL^−1^ for AsA, respectively. Interestingly, lowest values for both total phenols and DPPH scavenging were found in the Mr genotype, allowing a strong correlation index between the two parameters (R2 = 0.879).

### 3.6. Regression and Principal Component Analysis

**Figure 6:**
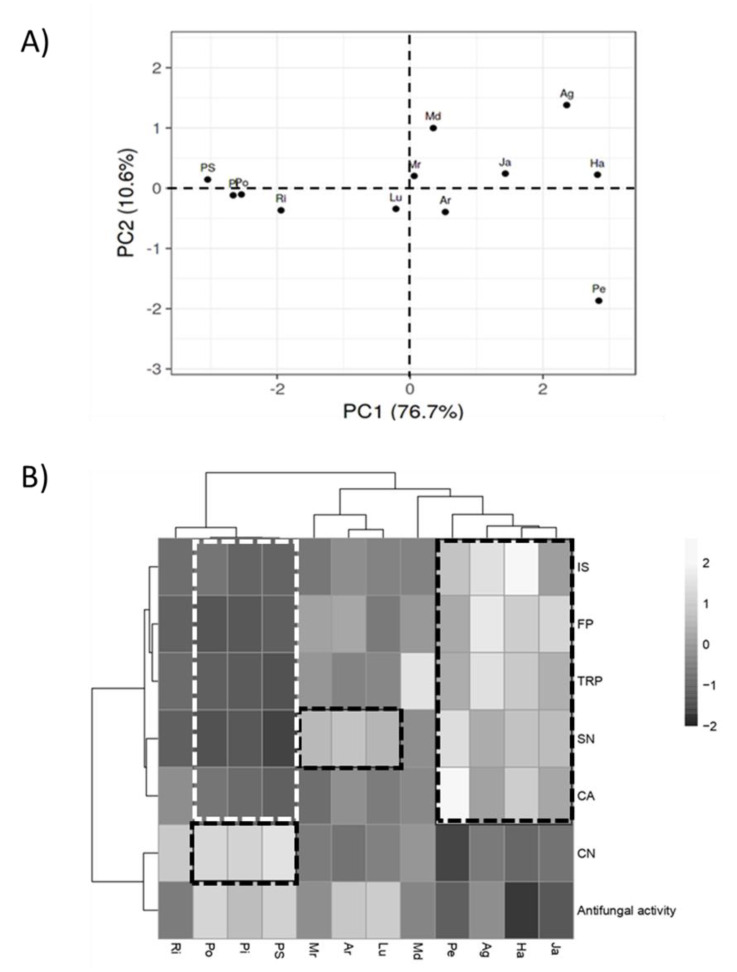
(**A**) Principal component analysis (PCA) of chemical compounds identified in potato cv Agria (Ag), AR-03-3410 (AR), Hansa (Ha), Jazzy (Ja), Luminella (Lu), Melody (Md), Melrose (Mr), Perline (Pe), Piatlina (Pi), Piccolo Star (PS), Postiglione (Po), and Ricciona di Napoli (Ri) foliar extracts. Unit variance scaling was applied to rows; SVD with imputation was used to calculate principal components. X and Y-axis show principal component 1 and principal component 2 that explain 76.7% and 10.6% of the total variance, respectively. (**B**) Hierarchical clustering of the extracts in relation with their antifungal activity and *n*-feruloylputrescine (FP), tryptophan (TRP) chlorogenic acid (CA), isoquercitrin (IS), α-chaconine (CN), α-solanine (SN) and solasodoside A (SDA) molecules. Rows were centered and unit variance scaling was applied. Both rows and columns were clustered using correlation distance and average linkage. Analysis was performed by ClustVis.

Principal component analysis (PCA) was conducted on identified chemical compounds to highlight significant metabolic variations across extracts. As reported in Figure 6A, significant differences were observed between the cluster including PS, Pi, Po, and Ri and all the other extracts along PC1. Using the loading plot to identify which variables have the largest effect on each component, we observed that CN and antifungal activity correlated positively with PC1, while a strong effect on PC2 was associated to CA. In order to determine the extent of differentiation among the extracts in relation with their antifungal activity, data were subjected to multiple variable analysis by cross-correlation matrix and then to hierarchical clustering (Figure 6B). The correlation matrix highlighted that antifungal activity was strongly affected by the abundance of CN (*p*-value < 0.05, R = 0.6), and was confirmed by hierarchical clustering analysis. In fact, this metabolite resulted predominant in Po, Pi and PS, which performed better in inhibit *R. solani* growth. In these samples, the abundance of IS, FP, TRP, SN, and CA was found lower than the other extracts.

On the other hand, extracts with mild antifungal activity (Ja, Ha, Ag, Pe, and Md) were characterized by high levels of IS, FP, TRP, SN, and CA. An exception was represented by Lu and AR extracts. In these cases, their appreciable antifungal activity was associated to low levels of CN but the abundance of SN was found significantly higher in them than in PS, Pi, and Po extracts. Furthermore, the level of all the other metabolites resulted meaningfully lower than Ja, Ha, Ag, Pe, and Md extracts, suggesting that the activity of each is strongly affected by its whole metabolite composition. 

**Figure 7:**
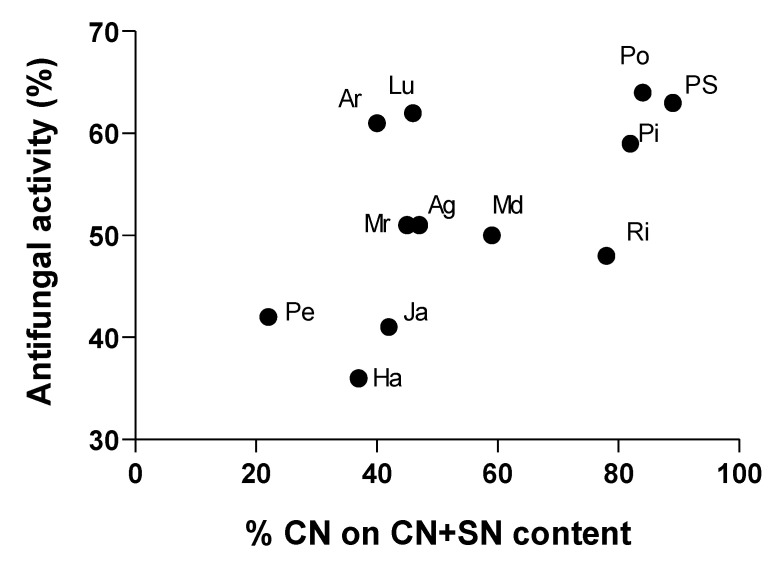
Pearson correlation analysis of the percentage of α-chaconine (CN) calculated on CN+ α-solanine (SN) content and antifungal activity of potato leaf cv Agria (Ag), AR-03-3410 (AR), Hansa (Ha), Jazzy (Ja), Luminella (Lu), Melody (Md), Melrose (Mr), Perline (Pe), Piatlina (Pi), Piccolo Star (PS), Postiglione (Po), and Ricciona di Napoli (Ri) foliar extracts.

Investigating deeply the relation between bioactivity and chemical features of extracts, we found that their antifungal activity highly correlated to the ratio between CN and SN. Indeed, as reported in Figure 7, extracts with a CN content around 50% or higher than 70% on the sum CN + SN, resulted in the most efficient treatments in terms of *R. solani* growth control.

## 4. Discussion

Plant extracts are a blend of bioactive molecules, which can work synergistically in counteracting harmful microorganisms [30,31]. In this study, the antifungal properties of leaf potato extracts were assessed both in vitro and in vivo on *R. solani*, revealing their potential applicability for the protection of rocket seedlings from damping-off disease occurrence. Five out of the tested phytochemical blends, showed high inhibitory activity on fungal growth, assessed in plate assays with EC50 values in the range of 3.5 and 14 mg mL^−1^. Moreover, two of them, Lu and Pe extracts, also showed a significant control efficacy in tray experiments. These findings agreed with a number of previous studies that have reported the efficacy of plant extracts against this pathogen. Damping-off disease on seeds and young seedlings is a major constraint of sowed crops against which several plant extracts have demonstrated promising protective capability when they are applied via seed treatments, soil drenching, or sprinkle [32,33]. On bean, for example, phytoextracts of various origin displayed encouraging reduction of Rhizoctonia root rot and damping-off symptoms [34,35]. On a set of winter vegetable species, garlic clove, and Allamanda leaf extracts were found effective in reducing both pre- and post-emergence damping-off after seed treatment by dipping [36], while on rice, *Clerodendrum*-chloroform extract was valid in controlling sheath blight disease [37].

LC–MS analysis conducted here putatively identified a set of possible antifungal compounds into the extracts, namely *n*-feruloylputrescine, chlorogenic acid, isoquercitrin, α-solanine, α-chaconine, solasodoside, and tryptophan. Moreover, *n*-feruloylputrescine is an amide of hydroxycinnamic acid with a presumable role in potato protection from phytopathogens [38,39]. It has been detected, recently, among the main molecular constituents of wild *Solanum* species methanolic leaf extracts exhibiting antibacterial, antiproliferative and antioxidant activity [40]. Chlorogenic acid and isoquercitrin are polyphenols commonly found in potato, associated to the antioxidant and antimicrobial activity [41,42]. These compounds have been previously reported as the major phenolic components of various antifungal plant extracts [43]. Polyphenolic compounds enrichment due to the solvent specificity, have associated to the strong antifungal activity of *Larrea tridentata*, *Flourensia cernua*, *Agave lechuguilla*, *Opuntia* sp. and *Yucca* sp. extracts against *R. solani* [44]. The main glycoalkaloids in potato, α-solanine, and α-chaconine showed a potent antimicrobial activity to a wide range of organisms; for this reason, they are considered as defensive allelochemicals [45]. On the basis of their fungal control potential, they are also formulated in silver nanoparticles as ecofriendly alternatives to conventional fungicides against different phytopathogens, including *R. solani* [46]. Among them, α-chaconine demonstrated higher antifungal activity compared to α-solanine [47,48]. In addition, it is well known that synergism between these two compounds enhances the membrane-disruptive activity of potato glycoalkaloid mixtures [49]. In particular, it was found that the toxicity of these mixtures is strictly dependent by the ratio between α-solanine and α-chaconine. As reported by Yamashoji et al. [50], a 1:1 ratio of these molecules was cytotoxic in three rat cell studies; while Roddick et al. [51] demonstrated a toxic effect on erythrocytes and fungal protoplasts with mixtures containing approximately 70% of α-chaconine. According with these, here, the extracts enriched in α-chaconine or α-solanine content resulted more effective in inhibit *R. solani* growth in vitro. Considering the two glycoalkaloids alone, the better performing phytochemical mixtures AR, Lu, PS, Pi, and Po were characterized by the α-chaconine content ranging between 46–89%. Solasodoside A is a steroidal glycosidic saponin that has been recently correlated to the antifungal activity of eggplant extracts against *Sclerotinia minor* [15]. The aromatic amino acid, tryptophan, has been reported as abundant constituent of antifungal peptides responsible of *Fusarium proliferatum* hyphal cells killing [52] and of plant defense metabolites [53]. 

Light microscopy experiments indicated that the exposure of the fungal mycelium to the two most in vivo-bioactive crude extracts, changed the normal morphology of hyphae undermining growth functions and viability of the pathogen. Accordingly, Khaledi et al. [54] have showed cytoplasmic coagulation and/or fragmentation in the *R. solani* hyphae treated with *Mentha piperita* and *Bunium persicum* essential oils. This suggests the occurrence of detrimental changes in the membrane functionality affecting feeding capability and the pathogen growth [55]. In our study, the observed translucency and cytoplasmic coagulation may indicate the occurrence of the cellular content leakage and cellular collapse as irremediable consequence of the plasma membrane disintegration affecting functionality and structural shape [56]. Accordingly, *Solanum* extracts have been seen to cause morphological alterations in *Curvularia lunata* consisting in reduced size and shape of conidia and marked hyphal septation [57]. 

Recently, a non-targeted metabolite profiling of potato leaves carried out using HPLC-ESI-QTOF-MS (high performance liquid chromatography coupled to quadrupole-time of flight mass spectrometry), tentatively identified 109 compounds, including organic acids, amino acids and derivatives, phenolic acids, flavonoids, iridoids, oxylipins and other polar and semi-polar compounds [26], demonstrating that they are an important source of bioactive molecules putatively responsible of the observed antifungal effects. Furthermore, these evidences support the suitability of these feedstock to be valorized as new products in a circular economy viewpoint. *S. tuberosum* peelings have previously investigated as potential source of new crude glycoalkaloid [58], polyphenolic [59], and polyphenolic/anthocyanin [60] antimicrobials. In addition, these natural compounds are characterized by a low persistence in the environment (the half-live of α-solanine has been estimated to vary in the range 1.8–4.1 days at 15 °C), features that makes them suitable for the field application in a perspective of ecofriendly control of plant disease. To our knowledge, this is the first report on the assessed antifungal effects of foliar potato extracts for their drench application in controlling rocket seedling damping-off.

## 5. Conclusions

In conclusion, potato phytochemical extracts have showed a great potential for plant protection applications in a perspective of developing sustainable agricultural practices with a big diversity of response among the assayed extracts from different genotypes, in a good relationship with the levels of glycoalkaloids and/or phenols. Findings of the current study suggested that the antifungal activity of the leaf extracts against *R. solani* may be explained by a direct action of phytochemicals against cell membrane integrity, as displayed by microscopy observations. Therefore, interestingly we found α-chaconine and α-solanine in a ratio compatible with high antifungal activity, let to hypothesize a role of these two glycoalkaloids in the pathogen control. However, we cannot exclude the contribution in the disease control mechanisms of other undetected metabolites. Future experiments are necessary to implement crop management protocols for the use at field-scale in replacing chemical fungicides, also with the use of synergic components. The suitability of these natural substances to be combined to antagonistic microorganisms, or other eco-friendly control mean under an integrated approach, are also desirable. An appropriate formulation coupled with an applicative method are further required to overcome interferences of the telluric microenvironment that may affect the antifungal performances of the biologically based molecules in field conditions [61].

## Figures and Tables

**Table 1:** EC50 of foliar potato cv Agria (Ag), AR-03-3410 (AR), Hansa (Ha), Jazzy (Ja), Luminella (Lu), Melody (Md), Melrose (Mr), Perline (Pe), Piatlina (Pi), Piccolo Star (PS), Postiglione (Po), and Ricciona di Napoli (Ri) extracts inhibiting *Rhizoctonia solani* mycelia growth.

Extract	EC50 (mg mL^−1^)
Ag	8.7
AR	10.1
Ha	>20
Ja	>20
Lu	3.4
Md	>20
Mr	>20
Pe	>20
Pi	12.7
Po	<5
PS	10.5
Ri	14.9

**Table 2:** Chlorogenic acid and α-solanine amounts in potato cv Agria (Ag), AR-03-3410 (AR), Hansa (Ha), Jazzy (Ja), Luminella (Lu), Melody (Md), Melrose (Mr), Perline (Pe), Piatlina (Pi), Piccolo Star (PS), Postiglione (Po), and Ricciona di Napoli (Ri) foliar extracts. Means are separated by Tukey HSD (honestly significant difference) test after ANOVA (*p* ≤ 0.05) and different lowercase letters indicate significant differences.

Extract	Chlorogenic Acid (µg mL^−1^)	α-Solanine (µg mL^−1^)
Ag	16.0 gh	205.2 h
AR	28.7 e	561.6 c
Ha	41.4 cd	385.6 e
Ja	15.0 h	200.3 h
Lu	19.2 f	492.4 d
Md	17.1 g	250.3 gh
Mr	7.4 i	324.0 ef
Pe	42.8 c	289.5 fg
Pi	39.6 d	685.6 ab
PS	15.2 h	347.8 ef
Po	72.8 b	749.5 a
Ri	83.7 a	631.0 b

**Table 3:** Identification and mean peak area of metabolites detected in potato cv Agria (Ag), AR-03-3410 (AR), Hansa (Ha), Jazzy (Ja), Luminella (Lu), Melody (Md), Melrose (Mr), Perline (Pe), Piatlina (Pi), Piccolo Star (PS), Postiglione (Po), and Ricciona di Napoli (Ri) foliar extracts.

Differential Metabolites	Retention Time (min)	*m/z* [M + H]^+^	Cultivar/Mean Peak Area ^a^											
			Ag	AR	Ha	Ja	Lu	Md	Mr	Pe	Pi	PS	Po	Ri
*n*-feruloylputrescine	0.96	265	259,690	350,220	341,280	196,770	199,220	209,140	204,680	154,370	329,850	335,740	365,070	357,190
Tryptophan	5.06	205	1,388,890	1,342,250	1,865,080	809,840	1,392,820	1,053,690	2,181,940	897,950	2,019,490	1,159,930	3,013,390	2,499,820
Chlorogenic Acid	7.97	355	625,420	1,121,230	1,615,650	585,590	749,440	288,190	668,030	1,669,850	1,547,820	591,670	2,843,520	3,266,750
Isoquercitrin	10.90	465	151,410	126,060	305,690	62,150	98,460	43,620	67,280	109,670	81,640	67,140	301,140	150,930
α-solanine	12.04	869	10,794,330	29,544,990	20,285,630	10,539,390	25,904,160	17,044,190	13,169,040	15,227,780	36,067,120	18,297,530	39,425,850	33,195,010
α-chaconine	12.20	852	9,445,350	19,896,460	12,034,560	7,576,490	22,501,550	13,829,460	18,599,510	4,338,610	165,521,180	149,545,270	211,153,460	118,210,830
Solasodoside	14.04	1031	278,860	5,380,510	908,010	279,170	628,970	11,197,310	572,650	611,320	598,380	121,228,390	625,520	377,770

^a^ Arbitrary scale

**Table 4:** Pearson coefficients resulting from cross-correlation between levels of biochemical compounds, chlorogenic acid (CA), solasodoside A (SDA), α-chaconine (CN), α-solanine (SN), isoquercitrin (IS), *n*-feruloylputrescine (FP), and tryptophan (TRP) detected in potato foliar extracts.

	CA	SDA	CN	SN	IS	FP	TRP
CA							
SDA	−0.27 ns						
CN	0.57 *	0.34 ns					
SN	0.72 **	−0.14 ns	0.74 **				
IS	0.62 **	−0.25 ns	0.27 ns	0.41 *			
FP	0.56 *	0.23 ns	0.67 **	0.69 **	0.55 *		
TRP	0.74 **	−0.25 ns	0.65 **	0.69 **	0.60 **	0.60 **	

Pearson coefficients resulting from cross-correlation between levels of biochemical compounds, chlorogenic acid (CA), solasodoside A (SDA), α-chaconine (CN), α-solanine (SN), isoquercitrin (IS), *n*-feruloylputrescine (FP), and tryptophan (TRP) detected in potato foliar extracts, * and ** significant for *p* < 0.05 and *p* < 0.01 respectively; ns: not significant.

## References

[1] Pane C., Sigillo L., Caputo M., Serratore G., Zaccardelli M., Tripodi P. (2017). Response of rocket salad germplasm (*Eruca* and *Diplotaxis spp*.) to major pathogens causing damping-off, wilting and leaf spot diseases. Arch. Phytopathol. Plant Prot..

[2] Lamichhane J.R., Dürr C., Schwanck A.A., Robin M.H., Sarthou J.P., Cellier V., Messéan A., Aubertot J.N. (2017). Integrated management of damping-off diseases. A review. Agron. Sustain. Dev..

[3] Ajayi-Oyetunde O.O., Bradley C.A. (2018). *Rhizoctonia solani*: Taxonomy, population biology and management of Rhizoctonia seedling disease of soybean. Plant Dis..

[4] Pandey A.K., Burlakoti R.R., Kenyon L., Nair R.M. (2018). Perspectives and challenges for sustainable management of fungal diseases of mungbean [*Vigna radiata* (L.) R. Wilczek var. radiata]: A Review. Front. Environ. Sci..

[5] Boulogne I., Petit P., Ozier-Lafontaine H., Desfontaines L., Loranger-Merciris G. (2012). Insecticidal and antifungal chemicals produced by plants: A review. Environ. Chem. Lett..

[6] Redondo-Blanco S., Fernández J., Lãpez-Ibáñez S., Miguélez E.M., Villar C.J., Lombã F. (2020). Plant phytochemicals in food preservation: Antifungal bioactivity: A review. J. Food. Prot..

[7] Derbalah A.S., Dewir Y.H., El-Sayed A.E.B. (2012). Antifungal activity of some plant extracts against sugar beet damping-off caused by *Sclerotium rolfsii*. Ann. Microbiol..

[8] El-Mougy N.S., Abdel-Kader M.M. (2007). Antifungal effect of powdered spices and their extracts on growth and activity of some fungi in relation to damping-off disease control. J. Plant Prot. Res..

[9] Abdel-Monaim M.F., Abo-Elyousr K.A.M., Morsy K.M. (2011). Effectiveness of plant extracts on suppression of damping-off and wilt diseases of lupine (*Lupinus termis* Forsik). Crop Prot..

[10] Aqil F., Ahmad I. (2003). Broad-spectrum antibacterial and antifungal properties of certain traditionally used Indian medicinal plants. World J. Microbiol. Biotechnol..

[11] Pane C., Fratianni F., Parisi M., Nazzaro F., Zaccardelli M. (2016). Control of Alternaria post-harvest infections on cherry tomato fruits by wild pepper phenolic-rich extracts. Crop Prot..

[12] Pane C., Francese G., Raimo F., Mennella G., Zaccardelli M. (2017). Activity of foliar extracts of cultivated eggplants against Sclerotinia lettuce drop disease and their phytochemical profiles. Eur. J. Plant Pathol..

[13] Pane C., Fratianni F., Raimo F., Nazzaro F., Zaccardelli M. (2017). Efficacy of phenolic-rich extracts from leaves of pepper landraces against Alternaria leaf blight of tomato. J. Plant Pathol..

[14] Esposito T., Celano R., Pane C., Piccinelli A.L., Sansone F., Picerno P., Zaccardelli M., Aquino R.P., Mencherini T. (2019). Chestnut (*Castanea sativa* Miller.) burs extracts and functional compounds: UHPLC-UV-HRMS profiling, antioxidant activity, and inhibitory effects on phytopathogenic fungi. Molecules.

[15] Rayne S., Karacabey E., Mazza G. (2008). Grape cane waste as a source of trans-resveratrol and trans-viniferin: High-value phytochemicals with medicinal and anti-phytopathogenic applications. Ind. Crop. Prod..

[16] Chomel M., Guittonny-Larchevêque M., Fernandez C., Gallet C., DesRochers A., Pare D., Jackson B.G., Baldy V. (2016). Plant secondary metabolites: A key driver of litter decomposition and soil nutrient cycling. J. Ecol..

[17] Mithen R., Raybould A.F., Giamoustaris A. (1995). Divergent selection for secondary metabolites between wild populations of Brassica oleracea and its implications for plant-herbivore interactions. Heredity.

[18] Hack H., Gall H., Klemke T., Klose R., Meier U., Stauss R., Witzenberger A. The BBCH-scale for phenological growth stages of potato (*Solanum tuberosum* L.). Proceedings of the 12th Annual Congress of the European Association for Potato Research.

[19] Pane C., Spaccini R., Piccolo A., Celano G., Zaccardelli M. (2019). Disease suppressiveness of agricultural greenwaste composts as related to chemical and bio-based properties shaped by different on-farm composting methods. Biol. Control.

[20] Brand-Williams W., Cuvelier M.E., Berset C. (1995). Use of a free radical method to evaluate antioxidant activity. LWT Food Sci. Technol..

[21] Foti M.C. (2015). Use and abuse of DPPH radical. J. Agric. Food Chem..

[22] Slinkard K., Singleton V.L. (1977). Total phenol analysis: Automation and comparison with manual methods. Am. J. Enol. Vitic..

[23] Docimo T., Francese G., Ruggiero A., Batelli G., De Palma M., Bassolino L., Toppino L., Rotino G.L., Mennella G., Tucci M. (2016). Phenylpropanoids accumulation in eggplant fruit: Characterization of biosynthetic genes and regulation by a myb transcription factor. Front. Plant Sci..

[24] Pushpa D., Yogendra K.N., Gunnaiah R., Kushalappa A.C., Murphy A. (2014). Identification of late blight resistance-related metabolites and genes in potato through nontargeted metabolomics. Plant Mol. Biol. Rep..

[25] Tai H.H., Worrall K., Pelletier Y., De Koeyer D., Calhoun L.A. (2014). Comparative metabolite profiling of Solanum tuberosum against six wild Solanum species with Colorado potato beetle resistance. J. Agric. Food Chem..

[26] Rodríguez-Pérez C., Gómez-Caravaca A.M., Guerra-Hernández E., Cerretani L., García-Villanova B., Verardo V. (2018). Comprehensive metabolite profiling of *Solanum tuberosum* L. (potato) leaves by HPLC-ESI-QTOF-MS. Food Res. Int..

[27] Wu S.B., Meyer R.S., Whitaker B.D., Litt A., Kennelly E.J. (2013). A new liquid chromatography-mass spectrometry-based strategy to integrate chemistry, morphology, and evolution of eggplant (*Solanum*) species. J. Chromatogr. A.

[28] Delledonne M., Dal Molin A., Minio A., Ferrarini A., Tononi P., Zamperin G., Toppino L., Sala T., Barchi L., Comino C. A high quality eggplant (*Solanum melongena* L.) genome draft and its use for mapping metabolic QTLs. Proceedings of the 11th Solanaceae Conference, SOL 2014.

[29] SAS Institute (2007). JMP Statistics and Graphics Guide.

[30] Palmer-Young E.C., Sadd B.M., Irwin R.E., Adler L.S. (2017). Synergistic effects of floral phytochemicals against a bumble bee parasite. Ecol. Evol..

[31] Zeng S.L., Duan L., Chen B.Z., Li P., Liu E.H. (2017). Chemicalome and metabolome profiling of polymethoxylated flavonoids in *Citri Reticulatae Pericarpium* based on an integrated strategy combining background subtraction and modified mass defect filter in a Microsoft Excel Platform. J. Chromatogr. A.

[32] Adandonon A., Aveling T.A., Labuschagne N., Tamo M. (2006). Biocontrol agents in combination with Moringa oleifera extract for integrated control of *Sclerotium*-caused cowpea damping-off and stem rot. Eur. J. Plant Pathol..

[33] Al-Askar A., Rashad Y. (2010). Efficacy of some plant extracts against *Rhizoctonia solani* on pea. J. Plant Prot. Res..

[34] Abd-El-Khair H., El-Gamal N.G. (2011). Effects of aqueous extracts of some plant species against *Fusarium solani* and *Rhizoctonia solani* in *Phaseolus vulgaris* plants. Arch. Phytopathol. Plant Prot..

[35] Mangang H.C., Chhetry G.K.N. (2012). Antifungal properties of certain plant extracts against *Rhizoctonia solani* causing root rot of French bean in organic soil of Manipur. Int. J. Sci. Res. Publ..

[36] Islam M.T., Faruq A.N. (2012). Effect of some medicinal plant extracts on damping-off disease of winter vegetable. World Appl. Sci. J..

[37] Choudhury D., Anand Y.R., Kundu S., Nath R., Kole R.K., Saha J. (2017). Effect of plant extracts against sheath blight of rice caused by *Rhizoctonia solani*. J. Pharmacogn. Phytochem..

[38] Malmberg A. (1984). N-Feruloylputrescine in infected potato tubers. Acta Chem. Scand. B.

[39] Macoy D.M., Kim W., Lee S.Y., Kim M.G. (2015). Biosynthesis, physiology, and functions of hydroxycinnamic acid amides in plants. Plant Biotechnol. Rep..

[40] Fadl Almoulah N., Voynikov Y., Gevrenova R., Schohn H., Tzanova T., Yagi S., Thomas J., Mignard B., Ahmed A.A.A., El Siddig M.A. (2017). Antibacterial, antiproliferative and antioxidant activity of leaf extracts of selected *Solanaceae* species. S. Afr. J. Bot..

[41] Thompson M.D., Thompson H.J., McGinley J.N., Neil E.S., Rush D.K., Holm D.G., Stushnoff C. (2009). Functional food characteristics of potato cultivars (*Solanum tuberosum* L.): Phytochemical composition and inhibition of 1-methyl-1-nitrosourea induced breast cancer in rats. J. Food Compos. Anal..

[42] Salawu S.O., Boligon A.A., Athayde M.L. (2014). Evaluation of antioxidant potential and nutritional values of white skinned sweet potato-unripe plantain composite flour blends. Int. J. Appl. Res. Nat. Prod..

[43] Koïta K., Neya F.B., Opoku N., Baissac Y., Campa C., Sankara P. (2017). Phytochemical analysis of Ziziphus mucronata Willd. extract and screening for antifungal activity against peanut pathogens. Afr. J. Plant Sci..

[44] Castillo F., Hernández D., Gallegos G., Mendez M., Rodríguez R., Reyes A., Aguilar C.N. (2010). In vitro antifungal activity of plant extracts obtained with alternative organic solvents against *Rhizoctonia solani* Kühn. Ind. Crops Prod..

[45] Friedman M., McDonald G.M. (1997). Potato glycoalkaloids: Chemistry, analysis, safety, and plant physiology. Crit. Rev. Plant Sci..

[46] Almadiy A.A., Nenaah G.E. (2018). Ecofriendly synthesis of silver nanoparticles using potato steroidal alkaloids and their activity against phytopathogenic fungi. Braz. Arch. Biol. Technol..

[47] Fewell A.M., Roddick J.G. (1993). Interactive antifungal activity of the glycoalkaloids α-solanine and α-chaconine. Phytochemistry.

[48] Fewell A.M., Roddick J.G. (1997). Potato glycoalkaloid impairment of fungal development. Mycol. Res..

[49] Smith D.B., Roddick J.G., Jones J.L. (2001). Synergism between the potato glycoalkaloids α-chaconine and α-solanine in inhibition of snail feeding. Phytochemistry.

[50] Yamashoji S., Matsuda T. (2013). Synergistic cytotoxicity induced by alpha-solanine and alpha-chaconine. Food Chem..

[51] Roddick J.G., Rijnenberg A.L., Osman S.F. (1988). Synergistic interaction between potato glycoalkaloids α-solanine andα-chaconine in relation to destabilization of cell membranes: Ecological implications. J. Chem. Ecol..

[52] López-García B., Harries E., Carmona L., Campos-Soriano L., López J.J., Manzanares P., Gandía M., Coca M., Marcos J.F. (2015). Concatemerization increases the inhibitory activity of short, cell-penetrating, cationic and tryptophan-rich antifungal peptides. Appl. Microbiol. Biotechnol..

[53] Consonni C., Bednarek P., Humphry M., Francocci F., Ferrari S., Harzen A., van Themaat E.V.L., Panstruga R. (2010). Tryptophan-derived metabolites are required for antifungal defense in the *Arabidopsis* mlo2 mutant. Plant Physiol..

[54] Khaledi N., Taheri P., Tarighi S. (2014). Antifungal activity of various essential oils against *Rhizoctonia solani* and *Macrophomina phaseolina* as major bean pathogens. J. Appl. Microbiol..

[55] Lee H.S., Kim Y. (2016). Antifungal activity of *Salvia miltiorrhiza* against *Candida albicans* is associated with the alteration of membrane permeability and (1,3)-β-D-glucan synthase activity. J. Microbiol. Biotechnol..

[56] Lengai G.M.W., Muthomi J.W., Mbega E.R. (2020). Phytochemical activity and role of botanical pesticides in pest management for sustainable agricultural crop production. Sci. Afr..

[57] Hernández-Rodríguez Z.G., Riley-Saldaña C.A., González-Esquinca A.R., Castro-Moreno M., de-la-Cruz-Chacón I. (2018). Antifungal activity of *Solanum* extracts against phytopathogenic *Curvularia lunata*. J. Plant Prot. Res..

[58] Yu J.O., Yap A.L., Tuason A.A., Tan C.C., Tan H.T., Tan L.V., Tan N.G., Tan R.T., Tangkusan D.I., Tiosin J.S. (2019). Antifungal activity of crude glycolated extracts of *Solanum tuberosum* L. (white potato) peelings against *Candida* and *Aspergillus* species. Acta Med. Philipp..

[59] Silveyra M.X., Lanteri M.L., Damiano R.B., Andreu A.B. (2018). Bactericidal and cytotoxic activities of polyphenol extracts from *Solanum tuberosum* spp. tuberosum and spp. andigena cultivars on Escherichia coli and human neuroblastoma SH-SY5Y cells in vitro. J. Nutr. Metab..

[60] De Masi L., Bontempo P., Rigano D., Stiuso P., Carafa V., Nebbioso A., Piacente S., Montoro P., Aversano R., D’Amelia V. (2020). Comparative phytochemical characterization, genetic profile, and antiproliferative activity of polyphenol-rich extracts from pigmented tubers of different *Solanum tuberosum* varieties. Molecules.

[61] Bowers J.H., Locke J.C. (2000). Effect of botanical extracts on the population density of *Fusarium oxysporum* in soil and control of Fusarium wilt in the greenhouse. Plant Dis..

